# Multi-tracer and multiparametric PET imaging to detect the IDH mutation in glioma: a preclinical translational in vitro, in vivo*,* and ex vivo study

**DOI:** 10.1186/s40644-022-00454-6

**Published:** 2022-03-18

**Authors:** Alexandra Clément, Timothee Zaragori, Romain Filosa, Olga Ovdiichuk, Marine Beaumont, Charlotte Collet, Emilie Roeder, Baptiste Martin, Fatiha Maskali, Muriel Barberi-Heyob, Celso Pouget, Matthieu Doyen, Antoine Verger

**Affiliations:** 1 Nancyclotep Molecular and Experimental Imaging Platform, CHRU-Nancy, 05 rue du Morvan, 54500 Vandoeuvre-Les-Nancy, France; 2 Lorraine University, INSERM, IADI UMR 1254, Nancy, France; 3 Lorraine University, CIC-IT UMR 1433, CHRU-Nancy, Nancy, France; 4 Lorraine University, CNRS, CRAN UMR 7039, Nancy, France; 5 Department of Pathology, CHRU-Nancy, Nancy, France; 6 Department of Nuclear Medicine, CHRU-Nancy, Nancy, France

**Keywords:** *IDH* mutation, PET, Gliomas, [^18^F]DPA-714

## Abstract

**Background:**

This translational study explores multi-tracer PET imaging for the non-invasive detection of the IDH1 mutation which is a positive prognostic factor in glioma.

**Methods:**

U87 human high-grade glioma (HGG) isogenic cell lines with or without the IDH1 mutation (CRISP/Cas9 method) were stereotactically grafted into rat brains, and examined, in vitro, in vivo and ex vivo*.* PET imaging sessions, with radiotracers specific for glycolytic metabolism ([^18^F]FDG), amino acid metabolism ([^18^F]FDopa), and inflammation ([^18^F]DPA-714), were performed sequentially during 3–4 days. The in vitro radiotracer uptake was expressed as percent per million cells. For each radiotracer examined in vivo, static analyses included the maximal and mean tumor-to-background ratio (TBR_max_ and TBR_mean_) and metabolic tumor volume (MTV). Dynamic analyses included the distribution volume ratio (DVR) and the relative residence time (RRT) extracted from a reference Logan model. Ex vivo analyses consisted of immunological analyses.

**Results:**

In vitro, IDH1+ cells (i.e. cells expressing the IDH1 mutation) showed lower levels of [^18^F]DPA-714 uptake compared to IDH1- cells (*p* < 0.01). These results were confirmed in vivo with lower [^18^F]DPA-714 uptake in IDH+ tumors (3.90 versus 5.52 for TBR_max_, *p* = 0.03). Different values of [^18^F]DPA-714 and [^18^F] FDopa RRT (respectively 11.07 versus 22.33 and 2.69 versus − 1.81 for IDH+ and IDH- tumors, *p* < 0.02) were also observed between the two types of tumors. RRT [^18^F]DPA-714 provided the best diagnostic performance to discriminate between the two cell lines (AUC of 100%, *p* < 0.01). Immuno-histological analyses revealed lower expression of Iba-1 and TSPO antibodies in IDH1+ tumors.

**Conclusions:**

[18F]DPA-714 and [18F] FDopa both correlate with the presence of the IDH1 mutation in HGG. These radiotracers are therefore good candidates for translational studies investigating their clinical applications in patients.

**Supplementary Information:**

The online version contains supplementary material available at 10.1186/s40644-022-00454-6.

## Background

The isocitrate dehydrogenase 1 (IDH1) mutation is a key molecular feature of the World Health Organization (WHO) Central Nervous System (CNS) tumor classification. This molecular alteration is a significant biomarker in the diagnostic and prognostic evaluation of gliomas [1, 2]. Gliomas expressing the IDH1 mutation are associated with better chemo- and radiotherapy responses and longer patient survival periods [2–4]. This IDH1 mutation leads to an intracytoplasmic accumulation of 2-hydroxyglutarate (2-HG) [5] which alters overall tumor metabolism [6].

Whilst several studies have used magnetic resonance imaging (MRI) to attempt to non-invasively characterize the IDH1 mutation in gliomas ([4, 7–12], the potential for positron emission tomography (PET) as a complementary metabolic imaging tool, is a growing field [13, 14]. PET imaging allows to depict different patho-physiological mechanisms to study changes in cellular metabolism. The diagnostic information acquired with ^18^F-fluorodeoxyglucose ([^18^F]FDG) allows to investigate glycolytic pathways albeit that this radiotracer is poorly suited to neuro-oncology applications owing to its high physiologic uptake by healthy brain tissue [14]. Amino acid PET tracers, such as ^18^F-fluoroethyltyrosine ([^18^F]-FET) or ^18^F-fluorodihydroxyphenylalanine ([^18^F]FDopa) however bind to the L-type amino acid transporter-1 (LAT-1) and may be used to specifically investigate amino-acid metabolism. These amino-acid PET radiotracers exhibit reduced uptake in healthy brain tissue, thereby enabling more accurate characterization of gliomas [15]. The [^18^F] FDopa radiotracer is widely used in the US and some European countries. It is cost-effective because, unlike ^18^F-FET, it can be used for other clinical neurology or oncology indications e.g., to detect parkinsonian disorders or neuroendocrine neoplasms [16]. Although IDH1 mutation status and its correlation with amino acid PET data remains ambiguous and heterogeneous [17], some recent analyses recommend using dynamic analyses for predicting the IDH mutation [18, 19]. New generation radiotracers have been developed to better characterize other glioma properties by PET imaging. Notable among these is the tumor inflammation specific [^18^F]DPA-714, a ligand of the translocator protein (TSPO), expressed in glioma cells [20–22]. It is this advance that has recently lead to reports of TSPO PET radiotracers as suitable options for the characterization of gliomas at initial diagnosis [23]. To the best of our knowledge, no study has to date combined multi-tracer static and dynamic PET acquisitions to investigate the metabolic changes induced by expression of the *IDH1* mutation in gliomas.

Currently, preclinical studies represent the sole means of investigating the *IDH1* mutation phenotype, independently of other mutations and tumor characteristics, in vivo. Previous preclinical studies have attempted to investigate the overexpression of the *IDH1* mutation in vivo [24–27] although none with the original objective of specifically investigating its pathophysiological expression by introducing the mutation in the well characterized U87 human high-grade gliomas (HGG) cell lines using CRISPR/Cas9. This innovative genetic approach allows to evaluate the effects of the isolated, basal expression of the *IDH1* mutation. We previously characterized in vivo physiologic and metabolic changes of the *IDH1* mutation using multiparametric MRI and spectroscopy [28]. Such multiple MRI sequences are nevertheless difficult to apply in clinical routine practice which underpins the recent interest in currently available PET radiotracers, particularly radiotracers specific for inflammation and metabolism, that may be able to inform about additional tumor properties and more targeted pathophysiological mechanisms.

The aim of our current study is to characterize in vitro and in vivo metabolic changes induced by the expression of the IDH mutation in U87 human derived HGG cell lines in the rat orthograft model, by using multiparametric and multitracer PET imaging specific for glycolytic metabolism ([^18^F]FDG), amino-acid metabolism ([^18^F]FDopa) and tumor inflammation ([^18^F]DPA-714) and corroborating this data with ex vivo immunological analyses.

## Methods

### U87-MG human derived HGG cell lines

U87 human derived HGG cell lines *IDH1*-mutated (IDH1+) and non *IDH1*-mutated (IDH1-) were purchased from the American Type Culture Collection® (ATCC, HTB-14IG and HTB-14). The two cells lines were cultured under standard conditions as previously described [28].

### Animal models

IDH1+ and IDH1- cells (5 × 10^4^ cells per animal) were implanted into the right caudate nucleus of the brain of athymic male nude rats (200–250 g; RH-Foxn1rnu; Envigo Gannat, France) as previously described [28].

After completing the imaging some of the anesthetized animals were sacrificed, by decapitation, for histological analysis (*N* = 20 IDH1+ and *N* = 14 IDH1-).

### Radiochemistry

[^18^F] FDG (FLUCIS®) and [^18^F] FDopa (DOPACIS®) were purchased from Curium (Nancy, FRANCE). [^18^F]DPA-714 radiosynthesis was implemented on the AIO® synthesizer to produce [^18^F]DPA-714 by nucleophilic substitution of the tosylated precursor OTs-DPA-714 (Pharmasynth, Estonia), as described in the literature [29].

### In vitro study

The IDH1+ and IDH1- U87 human derived HGG cells were resuspended in HBSS at a concentration of 2 × 10^5^/mL, and incubated (37 °C, 5% CO_2_/95% air) with 1 MBq/mL of each individual radiotracer for 30-, 60- and 120-min. Tracer uptake was stopped with the addition of ice-cold phosphate-buffered-saline (PBS). The cell substrate was then washed twice in cold PBS and resuspended in 1 ml of cold HBSS. The radioactivity, expressed as counts per minute and corrected for individual radiotracer decay, was measured using a calibrated gamma counter (Wizard, PerkinElmer®, France). Cell uptake, expressed as uptake per million cells, was determined by trypan blue. Individual radiotracer experiments were performed in duplicate for each of the three time points (*N* = 6) examined per radiotracer and per cell line.

### In vivo study

PET recordings were obtained with a camera dedicated to small animal studies (Inveon, Siemens Preclinical Solutions®, Knoxville, USA).

Two sets of rat brain acquisitions were performed, static acquisitions only for the first set of rats and dynamic and static acquisitions for the second set of rats.

**Fig. 1:**
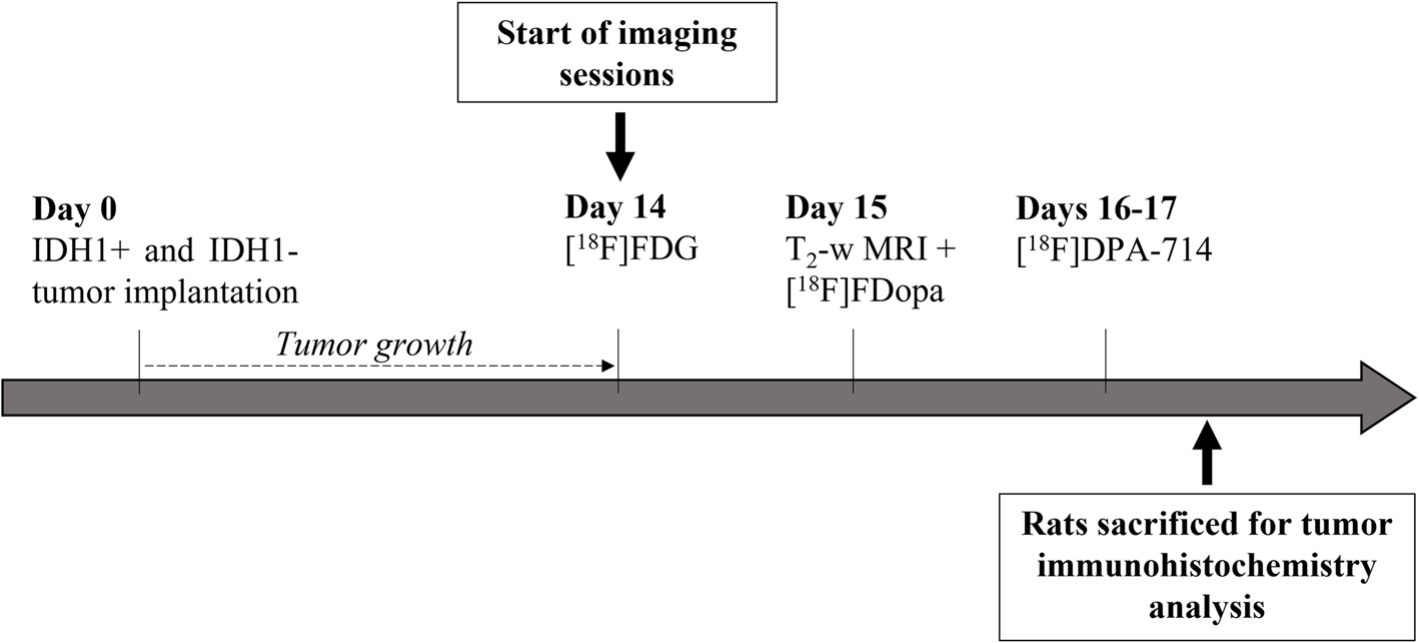
Flowchart of experimental design. Rats were orthotopically implanted with U87 human derived HGG IDH1+ or IDH1- cell lines. After tumor outgrowth, imaging sessions were performed sequentially during 3–4 days. The order in which radiotracers were imaged was predicated by their availability and was as follows [^18^F] FDG PET on the 14th day post tumor cell graft (p-g), MRI and [^18^F] FDopa on the 15th day p-g, [^18^F]DPA-714 between the 16th day and 17th day p-g. Rats were subsequently sacrificed for tumor histology analyses

A flowchart of the in vivo procedure is detailed in Fig. 1**.**

For static acquisitions, a 30-min PET scan was performed with [^18^F] FDG (injected activity of 74 MBq/mL) at 60 min p-i [30], [^18^F] FDopa (30 MBq/mL) at 30 min p-i [31] as recommended in the literature [32]. For [^18^F]DPA-714 radiotracer, a 20 min PET scan was acquired at 60 min p-i with an injected activity of 30 MBq/mL [33]. All PET images were reconstructed using the ordered-subset expectation maximization 3D algorithm (OSEM3D, 4 iterations, 16 subsets, zoom 1) together with scatter and attenuation corrections based on the transmission scan using a ^57^Co source measurement. The final voxel size was 0.77 × 0.77 × 0.79 mm^3^.

List-mode acquisitions were started a few seconds prior to the tracer injection with a 120 min acquisition for all radiotracers, and all acquired PET data were subsequently reconstructed in 27 consecutive frames (i.e., 5 frames × 120-s, 22 frames × 300-s). Reconstructed parameters were similar to those reported for static acquisitions.

MRI was performed on a 3Tesla scanner (Prisma, Siemens Healthineers®, Erlangen, Germany) with an 8-channel volume coil (Rapid Biomedical GmbH®, Rimpar, Germany). T_2_-weighted (T_2_-w) anatomical images were acquired using a Turbo Spin-Echo sequence (repetition time (TR)/echo-time (TE) = 2500/61 ms, voxel size = 255 × 255 × 1000 μm3, 24 slices, field of view (FOV) = 49 × 49 mm^2^, 8 averages) to define tumor volumes on the 15th day p-g.

Volumes of interest (VOIs) were defined on static PET and PET/T_2_-w MRI fused images with dedicated software (Inveon Research Workplace 4.1, Siemens®, Knoxville, USA) and centered on the maximal tumor uptake. From these VOIs, tumors VOIs were deduced from metabolic tumor volumes defined in the literature: with a 0.8 ratio of healthy brain tissue for [^18^F] FDG, a 1.3 ratio for [^18^F] FDopa [14], and a 1.8 ratio for [^18^F]DPA-714 [23]. The normal brain reference was defined as a symmetrical VOI in the contralateral healthy brain.

Maximum and mean Standardized Uptake Values (SUV_max_ and SUV_mean_, respectively) were determined from tumor VOIs. A partial volume effect correction (PVC) was applied to tumor SUV_max_ and SUV_mean_ values using the maximum and mean recovery coefficients (RCs) corresponding to the tumor volume, respectively. For each radiotracer, RCs were computed from phantom experiments using spherical inserts (volume range: [0.031 mL; 0.25 mL]) and a sphere-to-background contrast corresponding to the mean contrast observed between tumor and healthy brain. After PVC, tumor-to-normal-brain (TBR) ratios were computed as SUV_mean_ or SUV_max_ of the tumor uptake divided by the SUV_mean_ of the normal brain (TBR_mean_ and TBR_max_). VOIs previously defined on static images, were identically defined on dynamic images. For dynamic analyses, reference Logan models were generated for [^18^F] FDopa and [^18^F]DPA-714 as they have been shown to be adapted to the pharmacokinetics of these radiotracers [34, 35]. Two parameters, with regression starting at 30 min, were extracted from this model, namely the distribution volume ratio (DVR) and the relative residence time (RRT) computed respectively as the slope and the negative of the intercept. Additionally, the Dice Similarity Coefficient (DSC) [36] was further calculated at the individual rat level, to measure the index of similarity between different metabolic volumes obtained with different PET tracers.

The volume of interest (VOI) was manually delineated on the T_2_-w images of the MRIs of each rat brain to determine the tumor volume.

### Ex vivo study

Tumor tissue was fixed in 4% paraformaldehyde. Five-μm paraffin sections were incubated in 10 mM sodium citrate buffer (pH 6) for 20 min at 97 °C for dewaxing and antigen retrieval. Sections were stained with the following primary antibodies: anti-GLUT-1 (1:600, NB-22-3291, Neo Biotech), anti-CD-98 (anti-LAT-1) (1:500, LS-C4853000, LSBio), anti-Iba1 (1:100, anti-AIF1, ABIN2192043, antibodies-online.com) and anti-TSPO (1:200, BS9815M, Bioworld technology) along with Dako Autostainer Plus (Dako) and Flex+. All sections were examined by 2 observers (C.P. and A.C. or B.M.) on an Olympus BX 51 microscope and quantitative analysis was performed with the Image J image-processing software (Version 1.48). GLUT-1 hotspots were counted in one single light microscope field at 40x magnification. A staining score was applied to TSPO, Iba-1 and CD-98 with intensities defined as 0: no staining, 1: light, 2: moderate or strong with < 50% of cells staining, 3: strong with > 50% of cells staining.

### Statistical analyses

All analyses were performed using SPSS (IBM, SPSS Statistics version 25.0) and R version 4.1.1 (R Foundation for Statistical Computing, Vienna, Austria). The two-tailed significance level was set at *p* < 0.05. As the number of animals examined was not sufficient to assume a normal distribution, continuous variables were expressed as medians and interquartile ranges, except for in vitro variables which were expressed as means and standard deviations. PET and MRI parameters were compared using Mann Whitney with adjusted *p* values (Benjamini Hochberg correction) to reduce the false discovery rate. The ability of each individually extracted parameter to predict an IDH mutation was assessed using receiver operating characteristic (ROC) curves from which area under the curve (AUC), sensitivity, specificity and accuracy were computed. The optimal threshold was determined by selecting the point on the curve closest to (0,1).

## Results

### In-vitro study

**Fig. 2:**
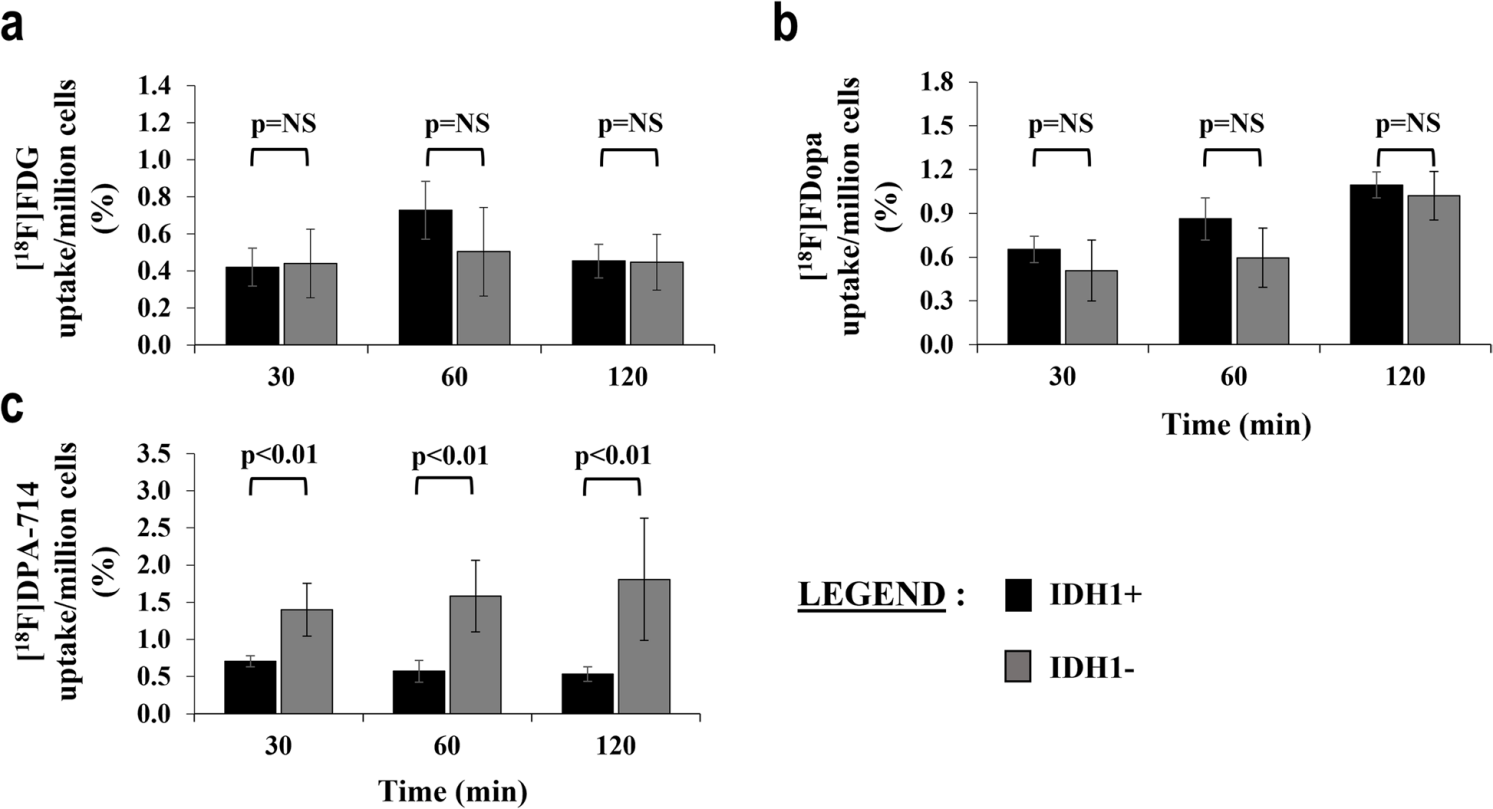
Histograms of in vitro multi-tracer analyses for [^18^F] FDG (a), [^18^F] FDopa (b) and [^18^F]DPA-714 (c) uptake in U87 human derived HGG IDH1+ and IDH1- cell lines after 30, 60, and 120 min incubation (*n* = 6 measurements for each individual tracer per time point). Radiotracer uptake was expressed as percent uptake per million tumor cells. Mann Whitney tests with *p* < 0.05 as significant

Figure 2 summarizes results of the in vitro study. There was no difference in cell uptake activity between IDH1+ and IDH1- tumors for [^18^F] FDG and the [^18^F] FDopa (*p* > 0.21). In contrast, IDH1+ cells exhibited significantly lower uptakes of [^18^F]DPA-714 at all concentration measurement time points when compared to IDH1- cells (0.71, 0.57, 0.53% of uptake/million cells at 30, 60, 120 min versus 1.40, 1.58 and 1.81% of uptake/million cells, *p* < 0.01).

### In-vivo study

#### Rat population

For static acquisitions, we induced 24 IDH1+ and 28 IDH1– rat brain tumors. For static and dynamic acquisitions of the second set of rats, 15 rats with IDH1+ and 14 rats with IDH1- tumors, were imaged. A detailed Table (Table S1) of available imaging session samples for each radiotracer with both static and dynamic datasets is presented in the additional file.

#### MRI and PET data analyses

**Fig. 3:**
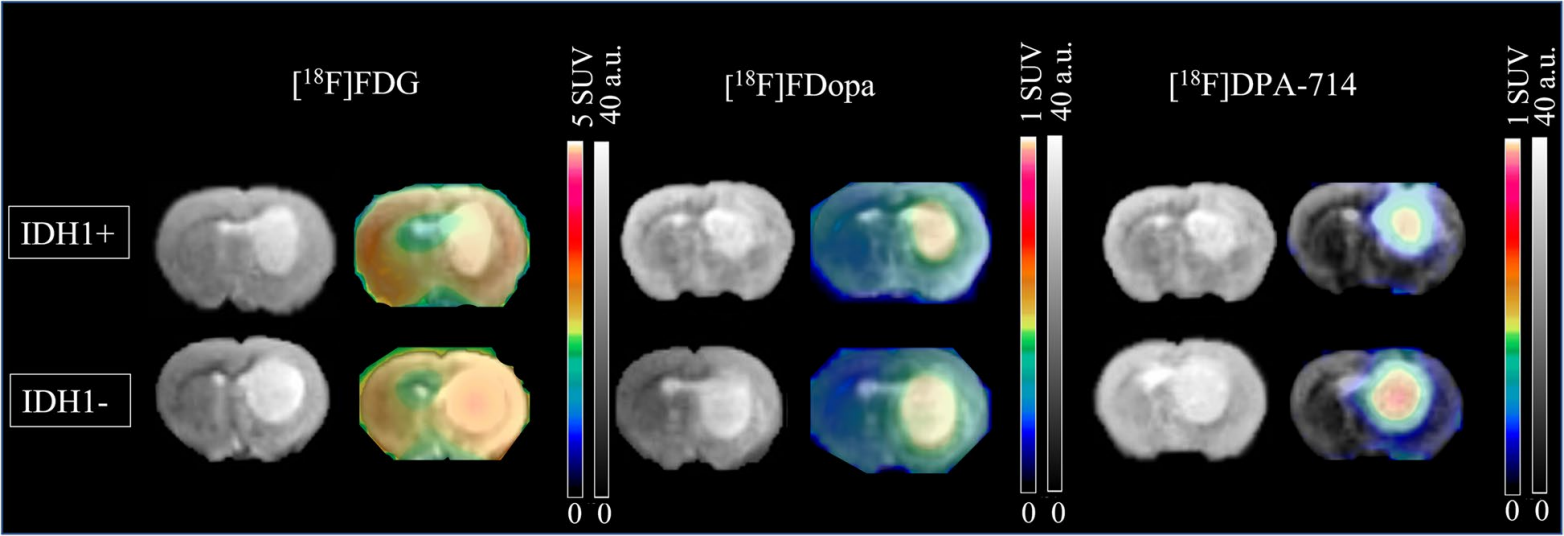
Representative axial images of T_2_-w MRI and static PET imaging merged with T_2_-w MRI for IDH1+ and IDH1- tumors with [^18^F] FDG, [^18^F] FDopa and [^18^F]DPA-714. MRI is expressed as signal intensity and PET on an SUV scale

**Fig. 4:**
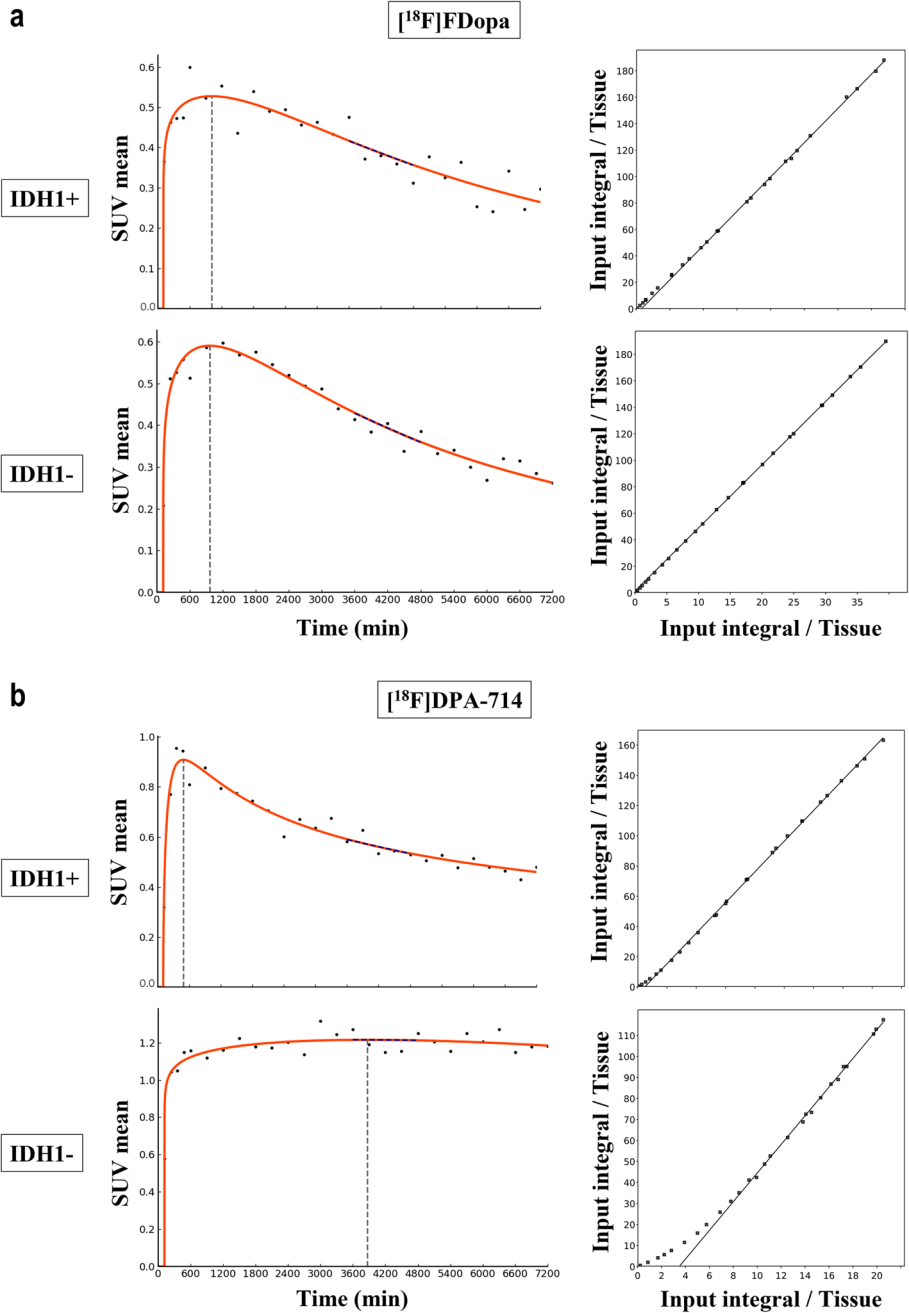
Representative examples of IDH+ and IDH- tumors with [^18^F] FDopa (a) and [^18^F]DPA-714 (b) dynamic acquisitions with time-activity curves (left panel) and the corresponding reference Logan models (right panel)

IDH1+ and IDH1– tumors grew to similar volumes, 23.35 [14.40; 66.25] mm^3^ versus 27.95 [18.00; 77.57] mm^3^, respectively (*p* = 0.83). PET results are summarized in Table 1. No significant difference between IDH1+ and IDH1- tumors was observed for [^18^F] FDG and [^18^F] FDopa static acquisitions (*p* ≥ 0.83). When compared to IDH1- tumors, IDH1+ tumors were however characterized by a significantly decreased [^18^F]DPA-714 TBR_max_ (3.90 [3.29; 4.67] vs.5.52 [4.72; 6.72], *p* = 0.03). In addition, the IDH1+ tumor characteristics were associated with significantly higher RRT in [^18^F] FDopa and lower RRT in [^18^F]DPA-714 (respectively 2.70 [1.45; 3.23] min for IDH+ versus − 1.81 [− 3.04; − 0.75] min for IDH-, *p* = 0.03 and 11.07 [7.09; 15.69] min for IDH+ versus 22.33 [20.68; 23.76] min for IDH-, *p* < 0.01). As displayed in Table 2, [^18^F]DPA-714 RRT provided the best diagnostic performances for identifying the IDH mutation (AUC of 1 [1;1]). Representative tumors imaged with MRI and multi-tracer PET are shown in Fig. 3. Examples of tumor activity curves and dynamic analyses are provided in Fig. 4.

**Table 1:** Results of univariate analyses of MRI and PET data. The Mann Whitney test was used to compare IDH1+ and IDH1- groups with significant, adjusted*p* values < 0.05 shown in bold

Static acquisitions									Dynamic acquisitions				
**Radio-tracers**	**IDH status**	**N**	**TBR**_**mean**_	**p**	**TBR**_**max**_	**p**	**MTV (mm**^**3**^**)**	**p**	**N**	**DVR**	**p**	**RRT**	**p**
**[**^**18**^**F] FDG**	**IDH1+**	*N* = 4	1.56 [1.46; 1.83]	0.91	1.37 [1.30; 1.54]	0.83	221.45 [176.60; 268.82]	0.96	–	–	–	–	–
	**IDH1-**	*N* = 7	1.69 [1.61; 1.75]		1.45 [1.43; 1.48]		250.00 [174.15; 273.25]		–	–		–	
**[**^**18**^**F] FDopa**	**IDH1+**	*N* = 13	2.86 [2.41; 3.36]	0.83	2.51 [2.07; 3.20]	0.83	29.00 [7.32; 220.70]	0.96	*N* = 8	2.63 [2.14; 3.74]	0.96	2.69 [1.45; 3.23]	**0.02**
	**IDH1-**	*N* = 13	2.57 [2.51; 2.95]		2.21 [2.11; 2.62]		72.00 [23.50; 202.00]		*N* = 6	2.57 [2.45; 3.52]		−1.81 [−3.04; −0.74]	
**[**^**18**^**F] DPA-714**	**IDH1+**	*N* = 7	4.48 [4.43; 4.67]	0.27	3.90 [3.29; 4.67]	**0.03**	48.50 [31.32; 56.60]	0.18	*N* = 7	5.28 [4.74; 5.49]	**0.05**	11.07 [7.08; 15.68]	**< 0.01**
	**IDH1-**	*N* = 8	4.86 [4.66; 5.16]		5.52 [4.72; 6.72]		76.80 [68.60; 113.20]		*N* = 8	9.03 [6.82; 9.73]		22.33 [20.68; 23.76]

**Table 2:** Diagnostic performances of significant parameters for identifying the IDH mutation in the univariate analysis

	AUC	Sensitivity	Specificity	Accuracy	Threshold
**TBR**_**max**_**[**^**18**^**F]DPA-714**	0.92	1	0.77	0.86	4.76
**RRT [**^**18**^**F]Dopa**	0.95	1	0.83	0.92	−0.44 min
**DVR [**^**18**^**F]DPA-714**	0.88	0.83	0.88	0.86	5.73
**RRT [**^**18**^**F]DPA-714**	1	1	1	1	18.4 min

#### Dice

The only exploitable results from the DICE coefficients (*N* ≥ 5 for direct comparisons between the two radiotracers) showed a low DICE coefficient between [^18^F] FDopa and [^18^F]DPA-714 (*n* = 9, 0.38 [0.26; 0.55]).

#### Post-sacrifice analyses

**Fig. 5:**
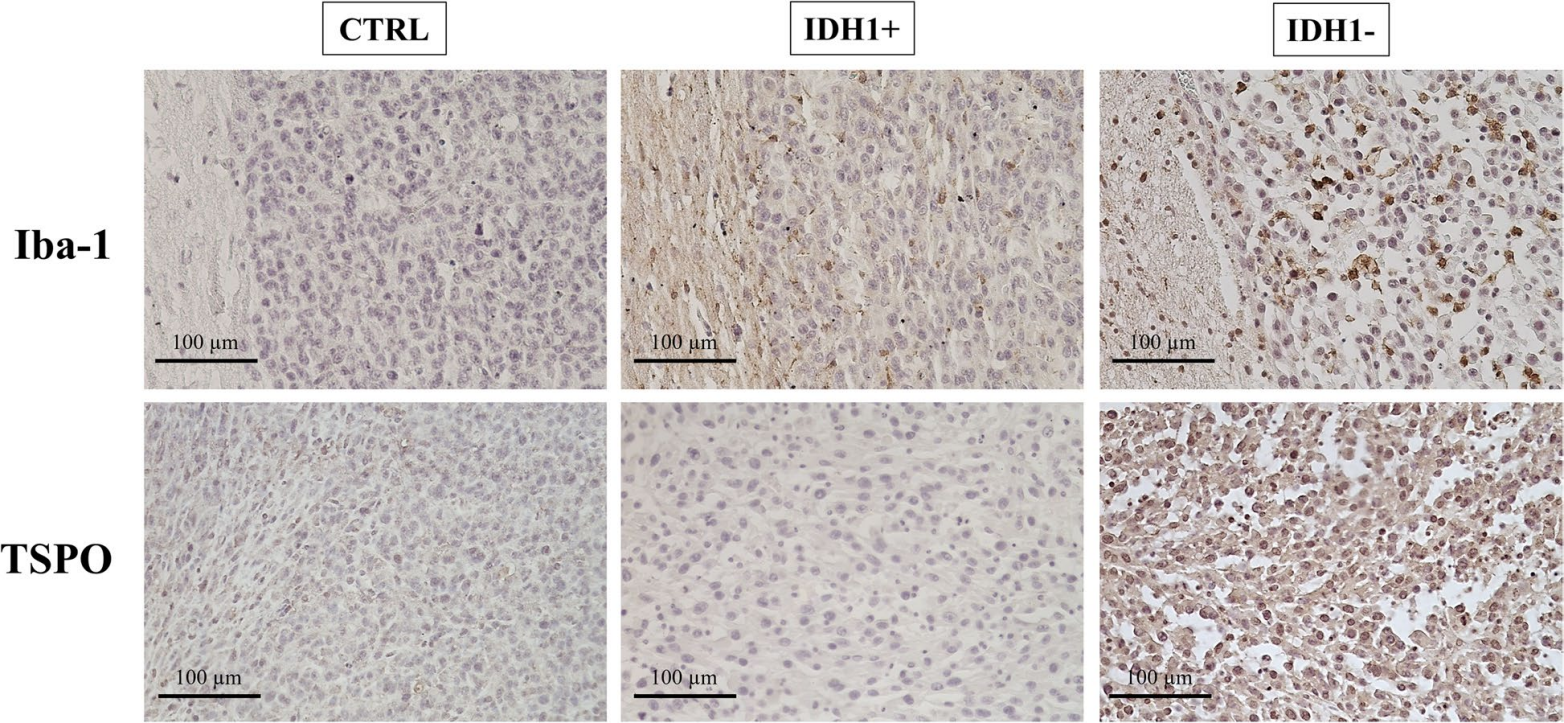
Representative IDH1+ and IDH1- and control rat brain immunohistochemistry tissue sections stained for Iba-1 (upper panels, magnification × 20) and TSPO (lower panels, magnification × 20). Scale 100 μm

There were no significant differences for GLUT-1, with hotspot median values of 28.00 [18.00; 38.00] for IDH1+ (*N* = 9) and 32.00 [26.75; 35.75] for IDH1- (*N* = 10), for LAT-1 staining with an average score of 2 for both tumor types (*N* = 7 IDH1+ and *N* = 11 IDH1-). However, Iba-1 and TSPO receptors showed lower staining in IDH+ compared to IDH- with respective average scores of 1.35 vs. 1.9 for Iba-1 and < 1 vs. 1.2 for TSPO (*N* = 3 IDH1+ and *N* = 5 IDH1-). Figure 5 shows a series of representative Iba-1 and TSPO staining patterns.

## Discussion

In this preclinical multiparametric and multi-tracer PET study, the *IDH1* mutation is associated with lower glioma uptake of [^18^F]DPA-714 in vitro, in vivo and ex vivo. In addition, the dynamic RRT [^18^F] FDopa parameter is also associated with the presence of the *IDH1* mutation. These preclinical results are consistent with recent results obtained in human patients [37, 38]. In contrast, no significant differences were observed between IDH1+ and IDH1- tumors for static [^18^F] FDG and [^18^F] FDopa acquisitions.

Our study used an animal model based on human derived HGG cell lines. Even though the *IDH* mutation is predominantly observed in glioma patients with low-grade tumors, one of the advantages of using a high-grade tumor model in preclinical studies is to reach a high enough rate of tumor growth potential in these models to accentuate any potential differences in uptake of the PET radiotracers investigated. Several experimental studies have endeveoured to develop candidate radiotracers for noninvasive PET imaging of the *IDH1* mutation but most of these yielded inconclusive results due to instability of the radiotracer in blood, lack of radiotracer selectivity or because experiments were not performed under representative physiologic conditions [39–41]. Our current study is original since it employs a combination of three different, easily clinically transposable, PET radiotracers to investigate specific pathophysiological pathways of glioma development that may be uniquely impacted by the *IDH1* mutation, using an innovative application of CRISPR/Cas9.

The radiotracer of interest from our current study is [^18^F]DPA-714, because it targets TSPO receptors which are upregulated during neuroinflammation. A lower uptake of this radiotracer was observed in IDH1+ tumors in vitro, in vivo, and ex vivo thus confirming the specificity of this radiotracer*.* The lower tracer uptake values are consistent with the reduced level of neuroinflammation and by extension the decreased aggressiveness of IDH+ tumors, which is in complete accordance with reports from the literature [38, 42]. Our recent study, which investigated multiparametric MRI, also found a spectroscopic profile of lower aggressiveness for IDH+ tumors, which further confirms results obtained in the current study [28]. Other mechanisms related to the IDH mutation may be suggested albeit based on assumptions that cannot be substantiated from our current data. In terms of metabolism, the IDH1 mutation may for instance lead to an intracytoplasmic accumulation of 2-hydroxyglutarate (2-HG) [5]. This may in turn alter the glioma epigenome by increasing DNA methylation [43, 44], activating cellular malignant transformation [45, 46], thereby altering overall tumor metabolism by inducing a downregulation of phospholipid biosynthesis [6, 47–49]. Such a mechanism may thus contribute to the less aggressive profile of tumors with an IDH mutation. Although amino-acid radiotracers have been increasingly studied in neuro-oncology [50], TSPO imaging seems to be particularly promising because it identifies glioma physio-pathological mechanisms that are distinct from those invoked by amino-acid tracers. This could explain the weak DICE index between TSPO and amino-acid uptake observed in our current study even though it was obtained from a small number of rats. TSPO imaging and IDH mutational status confer a trend towards higher ^18^F-GE-180-uptake in IDH-wild-type gliomas in the overall group, albeit that this trend was not confirmed in identical WHO grade gliomas [23]. Interestingly, both static and dynamic parameters for [^18^F]DPA-714 provided high predictive performance values to discriminate between the two tumor types, the best performances being obtained with the dynamic RRT [^18^F]DPA-714 (AUC of 1 [1;1]). Blood brain barrier (BBB) penetrance remains an issue for TSPO imaging [22] but is not a limitation for brain tumors in general or for gliomas per se since the brain blood barrier is disrupted in the vast majority of HGGs. Our previous study showed that contrast-enhancements were observed in our animal models, confirming that the BBB was disrupted [28]. In any case, our current study underlines the fact that using multiparametric PET, i.e. combining static and dynamic data, is very helpful to extract a maximum of information from PET imaging [37].

The second radiotracer of choice is the amino-acid PET [^18^F] FDopa radiotracer. Our current results are consistent with those obtained in human patients where dynamic [^18^F] FDopa PET parameters were the only factors that predicted the IDH mutation, whether it be compared to SUV-related or textural features [18, 51]. These previous results obtained in human patients could be influenced by multiple other histological or molecular cofounding factors. In our current study, the only difference between the two cell lines is the IDH mutation status. Since dynamic [^18^F] FDopa PET parameters were associated with the IDH mutation, unlike any of the in vitro or ex vivo variables, it appears that dynamic parameters predominantly detect perfusion phase differences between the IDH1+ and IDH1- cell types. Indeed, wash-in-wash-out phases are typically observed in aggressive IDH-wildtype gliomas, which may correspond to tumors that not only express high concentrations of LAT transporters but also tumors that are characterized by more extensive tracer perfusion as discussed in our previous publication [34]. In contrast, our [^18^F] FDG PET results are somewhat less surprising, and consistent with reports in the literature, since this radiotracer does not allow to differentiate tumor tissue from healthy brain cortex because of its physiologic uptake in healthy brain tissue, which limits the potential usefulness of this radiotracer in neuro-oncology [13] .

Our study does have several inherent limitations. Even though our tissue staining results confirmed the PET results, neo-angiogenesis and microglia/macrophages (GAMs), may have contributed to the TSPO signal [22]. This was not specifically tested in our current study. Further preclinical studies with post-autopsy histo-radiography analyses are required to better define the specific regions of radiotracer uptake. Moreover, it is known that TSPO polymorphisms can alter radiotracer uptake, which would limit the radiotracer’s potential usefulness in translational human studies. Our current study is also limited by the fact that not all rats were able to be PET imaged for all three radiotracers, due to the differing availabilities of individual radiotracers delivered to the lab at the time. In addition, although the volumes of IDH1+ tumors were statistically similar to IDH1- tumors, tumor growth varied between the groups and between radiotracer acquisitions. The concordance of in vitro, in vivo and ex vivo results at least for the [^18^F]DPA-714 PET nevertheless suggests that the contribution of any such potentially confounding effects is limited.

## Conclusion

To summarize, [^18^F]DPA-714 and [^18^F] FDopa are two PET radiotracers that are associated with the presence of the *IDH1* mutation in HGG in this preclinical study. Their complementarity may lead to future translational studies exploring their clinical applications in patients.

## Supplementary Information

**Additional file 1.**

## Data Availability

The datasets used and/or analyzed during the current study are available from the corresponding author on reasonable request.

## Abbreviations

AUC: Area under the curve
CNS: Central nervous system
DSC: Dice similarity coefficient
DVR: Distribution volume ratio
FOV: Field of view
HGG: Human high-grade glioma
IDH: Isocitrate dehydrogenase
LAT-1: L-type amino acid transporter-1
MRI: Magnetic resonance imaging
MTV: Metabolic tumor volume
PBS: Phosphate-buffered-saline
PET: Positron emission tomography
PVC: Partial volume effect correction
RCs: Recovery coefficients
ROC: Receiver operating curves
RRT: Relative residence time
SUV_max_: Maximum standardized uptake values
SUV_mean_: Mean standardized uptake values
TBR_max_: Maximal tumor-to-background ratio
TBR_mean_: Mean tumor-to-background ratio
TE: Echo-time
TR: Repetition time
TSPO: Translocator protein
VOI: Volume of interest
WHO: World health organization
