# Supplementary material for: Multi-tracer and multiparametric PET imaging to detect the IDH mutation in glioma: a preclinical translational in vitro, in vivo, and ex vivo study
Source: Cancer Imaging. 2022 Mar 18;22:16. doi: 10.1186/s40644-022-00454-6 (PMC8932106; doi:10.1186/s40644-022-00454-6)
Supplement: Supplementary file 1 — Additional file 1. [file 40644_2022_454_MOESM1_ESM.docx]

| **Static** | | | | | | | |
| --- | --- | --- | --- | --- | --- | --- | --- |
| **Radiotracers** | **Radiotracers** | **[^18^F]FDG** | | **[^18^F]FDopa** | | **[^18^F]DPA-714** | |
|  | **IDH1 status** | **IDH1+** | **IDH1-** | **IDH1+** | **IDH1-** | **IDH1+** | **IDH1-** |
| **[^18^F]FDG** | **IDH1+** | **4** | **-** | 4 | - | 0 | - |
|  | **IDH1-** | **-** | **7** | - | 7 | - | 0 |
| **[^18^F]FDopa** | **IDH1+** | 4 | - | **13** | **-** | 5 | - |
|  | **IDH1-** | - | 7 | **-** | **13** | - | 5 |
| **[^18^F]DPA-714** | **IDH1+** | 0 | - | 5 | - | **7** | **-** |
|  | **IDH1-** | - | 0 | - | 5 | **-** | **8** |
| **Static and dynamic** | | | | | | | |
| **[^18^F]FDopa** | **IDH1+** | 0 | - | **8** | **-** | 5 | - |
|  | **IDH1-** | - | 0 | **-** | **6** | - | 5 |
| **[^18^F]DPA-714** | **IDH1+** | 0 | - | 5 | - | **7** | **-** |
|  | **IDH1-** | - | 0 | - | 5 | **-** | **8** |

**Table S1** Two-way table of available imaging session samples for each radiotracer with both static and list-mode acquisitions**.**
